# Methotrexate-Induced Alteration of Renal Aquaporins 1 and 2, Oxidative Stress and Tubular Apoptosis Can Be Attenuated by Omega-3 Fatty Acids Supplementation

**DOI:** 10.3390/ijms232112794

**Published:** 2022-10-24

**Authors:** Mosaab Salah El-din El-Agawy, Alaa Mohamed Mohamed Badawy, Mohammed R. Rabei, Mohamed Mahmoud Abdelraheem Elshaer, Eman Mohamad El Nashar, Mansour A. Alghamdi, Mohammed A. Alshehri, Hassan Reda Hassan Elsayed

**Affiliations:** 1 Department of Anatomy and Embryology, Faculty of Medicine, Mansoura University, Mansoura 35516, Egypt; 2 Department of Anatomy, Faculty of Medicine, New Mansoura University, New Mansoura 35712, Egypt; 3 Department of Medical Physiology, Faculty of Medicine, Mansoura University, Mansoura 35516, Egypt; 4 Department of Physiology, Faculty of Medicine, King Salman International University, South Sinai 46511, Egypt; 5 Department of Clinical Pharmacology, Faculty of Medicine, Ain Shams University, Cairo 11566, Egypt; 6 Department of Clinical Pharmacology, Faculty of Medicine, King Salman International University, South Sinai 46511, Egypt; 7 Department of Anatomy, College of Medicine, King Khalid University, Abha 61421, Saudi Arabia; 8 Department of Histology and Cell Biology, Faculty of Medicine, Benha University, Benha 13511, Egypt; 9 Genomics and Personalized Medicine Unit, College of Medicine, King Khalid University, Abha 61421, Saudi Arabia; 10 Nephrology Section, Internal Medicine Department, College of Medicine, King Khalid University, Abha 61421, Saudi Arabia

**Keywords:** methotrexate, kidney, omega-3 fatty acids, apoptosis, aquaporins, oxidative stress

## Abstract

Methotrexate (MTX) is a potent anti-cancer drug, commonly associated with nephrotoxicity via the induction of oxidative stress and apoptosis with alteration of renal water channel proteins, namely aquaporins (AQPs). Omega-3 long-chain polyunsaturated fatty acids (LC-PUFA) have shown cytoprotective effects through their anti-oxidant and antiapoptotic activities. The present study aims for the first time to explore the role of LC-PUFA against MTX-induced nephrotoxicity. Rats were divided into the following groups: saline control, LC-PUFA control, MTX, MTX + LC-PUFA (150 mg/kg), or MTX + LC-PUFA (300 mg/kg). Then, H&E staining and immunohistochemical staining for the anti-apoptosis marker B-cell lymphoma 2 (BCL-2), the apoptosis marker BCL2-Associated X Protein (BAX), the proinflammatory marker Nuclear factor kappa B (NF-kB), AQPs 1 and 2 were performed in kidney sections with an assessment of renal oxidative stress. The MTX caused a renal histopathological alteration, upregulated renal BAX and NF-kB, downregulated Bcl-2 and AQP1, altered the distribution of AQP2, and caused oxidative stress. The LC-PUFA attenuated the pathological changes and decreased renal BAX and NF-kB, increased BCL-2 and AQP1, restored the normal distribution of AQP2, and decreased the oxidative stress. Therefore, LC-PUFA is a good adjuvant to MTX to prevent its adverse effects on kidneys through its antiapoptotic, antioxidant, and anti-inflammatory effect and its role in the restoration of the expression of AQPs 1 and 2.

## 1. Introduction

Methotrexate (MTX) is commonly prescribed for the management of malignant tumors [1] and autoimmune disorders, such as rheumatoid arthritis and psoriasis [2,3]. As a classical folate antagonist, MTX inhibits the dihydrofolate reductase enzyme [4,5]. This results in interruption of DNA replication, ATP synthesis, suppression of cell division, and probable apoptosis [6]. The MTX does not only affect cancer cells, but can affect normal cells also [7]. Thus, its clinical use is restricted, as it can cause a damage to bone marrow, the liver, kidneys, lungs, and intestines [8]. 

Since MTX is mainly excreted by the kidneys by glomerular filtration and active transport, nephrotoxicity is a usual adverse effect [9]. The mechanism by which MTX induces nephrotoxicity is not clear; however, induction of oxidative stress, suppression of DNA production, inflammatory infiltration, and apoptosis may play important roles [10,11]. Thus, searching for a nephroprotective drug to decrease nephrotoxicity induced after MTX is essential [7].

Aquaporins (AQPs) are transmembrane water channel proteins that regulate urine concentration, osmolarity, and volume of fluid. The epithelial lining of the kidney is the main site for transporting fluids. The kidney has seven types of AQPs present in the different parts of nephrons [12]. The main station for the absorption of most fluids, after their passage through glomeruli, is the proximal convoluted tubule, where AQP1 is strongly expressed [13]. On the other hand, AQP2 is mainly present in the epithelial cells of collecting ducts which have an important role in the concentration of urine and body fluid volume control. The AQP2 is controlled by vasopressin. [14]. Aquaporin expression and distribution are adversely affected by MTX [15,16,17].

Omega-3 essential polyunsaturated fatty acids include docosahexaenoic acid (DHA), alpha-linoleic acid, and eicosapentaenoic acid (EPA). They are obtained from fatty fish, seafood, walnuts, and soy [18]. They are mainly obtained from the diet because they cannot be synthesized in adequate quantities by the human body [19]. Furthermore, LC-PUFAs proved efficient in protecting against, as well as treating, many diseases through their antioxidant and anti-inflammatory activities [20,21,22,23]. Moreover, LC-PUFAs could protect against apoptosis, induced by MTX in the spleen [24] and intestinal mucosa [25], as well as against lipopolysaccharide-induced acute kidney injury [26]. Arab et al. proved the protective effect of camel milk on methotrexate-induced kidney injury [27]. This protective effect may be attributed in part to the high content of PUFA (30%) in camel milk. Thus, the present study aimed to clarify the role of LC-PUFA, in its pure form, against MTX-induced nephrotoxicity.

**Figure 1:**
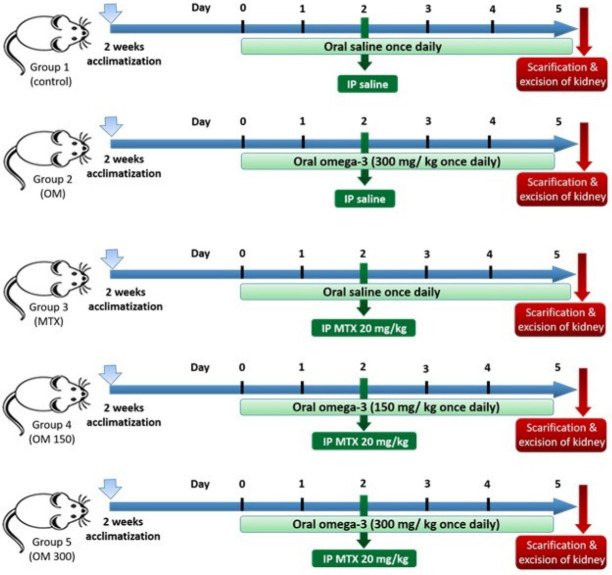
The timeline of the study.

We have performed this study to investigate the efficacy of LC-PUFA supplementation on acute nephrotoxicity, induced by methotrexate, as well as to study its antiapoptotic and anti-inflammatory roles, and its role in the regulation of the expression of aquaporins 1 and 2. The timeline for the study is demonstrated in Figure 1.

## 2. Results

### 2.1. Effect of LC-PUFA on MTX-Induced Alteration of Body Weight and on Renal Function Tests

**Figure 2:**
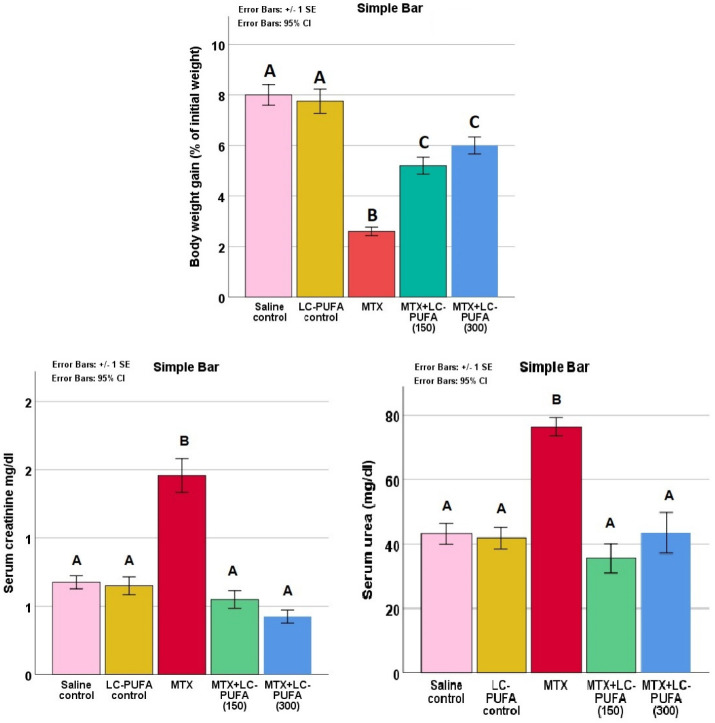
Serum creatinine and urea. Data are presented as mean ± standard error. Different letters = significant difference.

The study of body weight gain, serum creatinine, and urea in the study groups expressed a significant difference (*p* < 0.0005 for all, F value = 36.918, 29.242 and 14.421 for body weight, creatinine, and urea, respectively). The Tukey post-hoc test showed significant elevation in serum creatinine and urea, as well as a significant reduced weight gain in the MTX group compared to the control groups. The MTX + LC-PUFA groups showed a significant decrease in creatinine and urea and a significantly higher weight gain, compared to the MTX group (Figure 2). 

### 2.2. Effect of LC-PUFA on Oxidative Stress Markers in MTX-Treated Rats

**Figure 3:**
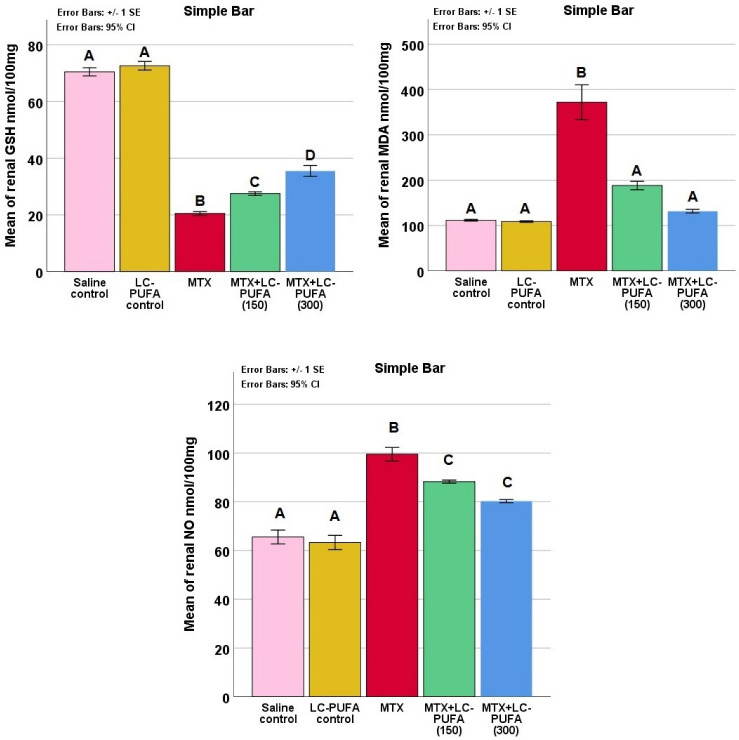
Renal oxidative stress markers. Data are presented as mean ± standard error. Different letters = significant difference. Here, GSH is reduced glutathione, NO is nitric oxide, and MDA is malonaldehyde.

The study of the renal oxidative stress markers reduced glutathione (GSH), malonaldehyde (MDA), and nitric oxide (NO) in the study groups showed significant differences (*p* < 0.0005 for all, F value = 337.899, 38.836, and 46.505 for GSH, MDA, and NO, respectively). The Tukey post-hoc test showed a significant elevation in renal MDA and NO as well as a significant reduction in GSH in the MTX group, compared to the control groups. The MTX + LC-PUFA groups showed a significant decrease in MDA and NO as well as a significant elevation in GSH, as compared to the MTX group. There was a significant elevation in GSH in MTX + LC-PUFA (300), compared to MTX + LC-PUFA (150). The MTX + LC-PUFA groups still show a significant difference from the control group except for MDA which showed no significant difference between them (Figure 3).

### 2.3. Effects of LC-PUFA on MTX-Induced Renal Histopathological Alteration and Histopathological Scoring

**Figure 4:**
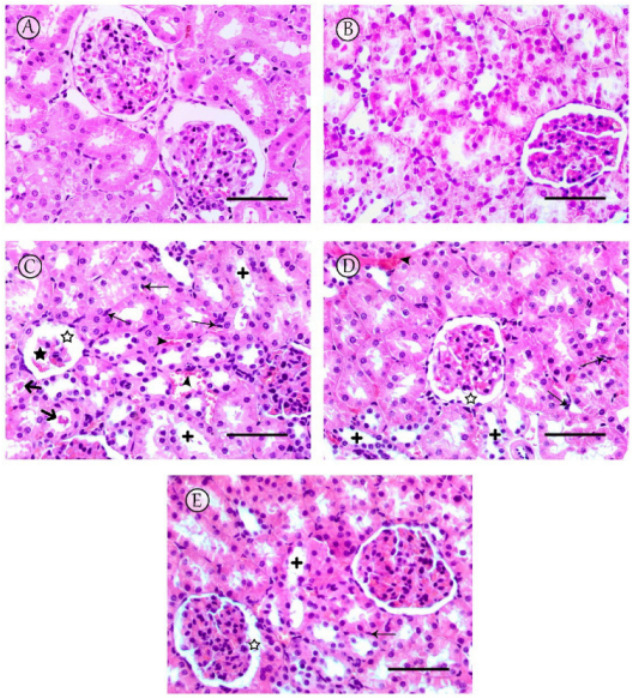
Histopathological changes observed in kidneys. (**A**,**B**) Sections from saline and LC-PUFA control groups revealed normal renal glomeruli and tubules. (**C**) The MTX group revealed severe damage to the renal tissue, particularly in the renal cortex, including shrinkage of the glomeruli (star) with dilatation of the Bowman’s capsule (white star), tubular dilatation (+) with occasional tubule cast presence (thick arrow), and inflammatory cell infiltrations (thin arrows), with congestion of renal blood vessels (arrowheads). (**D**) The MTX + LC-PUFA 150 group showed moderate dilatation of tubules (+) with dilatation of the Bowman’s capsule (white star), and inflammatory cell infiltrations (thin arrows) with congestion of renal blood vessels (arrowheads). (**E**) The MTX + LC-PUFA 300 group observed maximum improvement in cytoarchitectural changes with no hemorrhage, minimal tubular dilatation, minimal inflammatory cell infiltrations (thin arrows), and dilatation of the Bowman’s capsule (white star). Scale bar = 50 µm.

Kidneys of the rats of the saline control and LC-PUFA groups showed normal renal glomeruli and tubules (Figure 4A,B). However, the renal cortices of the MTX group showed histopathological changes, including shrinkage of the glomeruli, dilatation of the Bowman’s capsule, tubular dilatation, occasional tubule cast presence, inflammatory cell infiltrations, and congestion of renal blood vessels (Figure 4C). The MTX + LC-PUFA (150 mg/kg) group revealed mild dilatation of the renal tubules, dilatation of the Bowman’s capsule, and inflammatory cell infiltrations with congestion of renal blood vessels (Figure 4D). A notable attenuation of the pathological changes was revealed in MTX + LC-PUFA (300 mg/kg) group, in which the glomeruli appeared nearly normal with mild dilatation in the Bowman’s capsule and renal tubules and few inflammatory cells (Figure 4E). The low power ((×100 magnification) is demonstrated in Figure S1. The histopathological score among the groups showed a significant difference with higher scores in the MTX group (median value = 10) when compared to the control groups (median value = 1), with significant reduction in the LC-PUFA 300 groups (median values = 4) when compared to the MTX group (Table 1).

### 2.4. Effect of LC-PUFA on Immunohistochemical Analysis of NF-ĸB, BAX, Bcl2, AQP1, and AQP2

**Figure 5:**
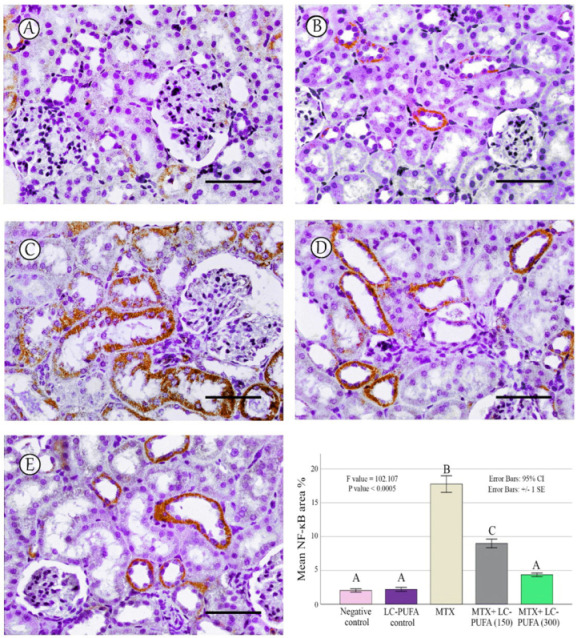
NF-ĸB immunostaining; (**A**): Saline group; (**B**): LC-PUFA group; (**C**): MTX group; (**D**): MTX + LC-PUFA 150 group; (**E**): MTX + LC-PUFA 300 group. Results revealed minimal positive staining in the kidneys of the control and LC-PUFA group. However, strong diffuse expression was demonstrated in the MTX-treated rats. Moreover, the epithelium of the renal tubules of MTX + LC-PUFA 150 and MTX + LC-PUFA 300 groups showed a weak reaction. Scale bar = 50 µm. The histogram showsarea fraction of NF-ĸB. Results are introduced as mean ± standard error. The *p* value is cited as letters (different letters mean a significant difference).

**Figure 6:**
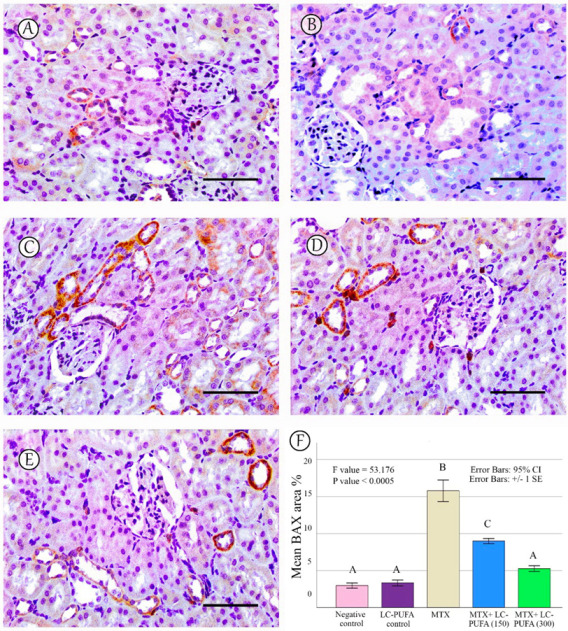
BAX immunostaining. (**A**): Saline group; (**B**): LC-PUFA group; (**C**): MTX group; (**D**): MTX + LC-PUFA 150 group; (**E**): MTX + LC-PUFA 300 group. Results revealed minimal positive staining in the saline and LC-PUFA groups. However, strong diffuse expression was demonstrated in the MTX-treated rats. Moreover, the epithelium of the renal tubules of the MTX + LC-PUFA 150 and MTX + LC-PUFA 300 groups showed a weak focal reaction. Scale bar = 50 µm. (**F**) Area fraction of BAX positive area. Results are introduced as mean ± standard error. The *p* value is cited as letters (different letters mean a significant difference).

**Figure 7:**
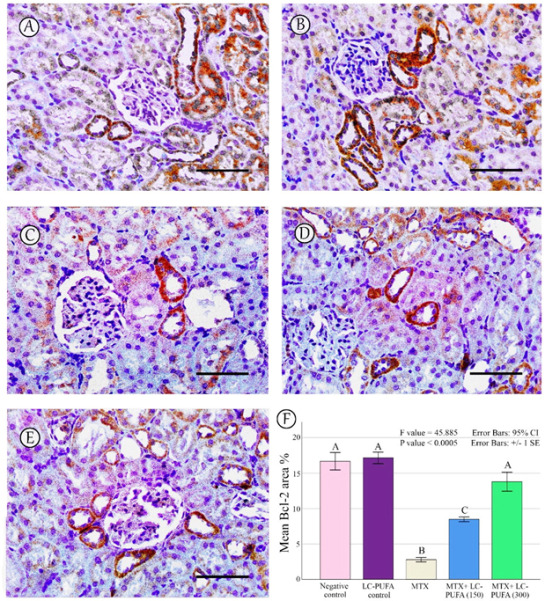
Bcl-2 immunostaining; (**A**): Saline group; (**B**): LC-PUFA group; (**C**): MTX group; (**D**): MTX + LC-PUFA 150 group; (**E**): MTX + LC-PUFA 300 group. Results revealed a moderate expression in the renal tubular epithelium of the control and LC-PUFA group rats. The expression was mild in the MTX-treated rats. However, the renal tubular epithelium of MTX + LC-PUFA 150 and MTX + LC-PUFA 300 groups showed moderate Bcl2 expression. Scale bar = 50 µm. (**F**) Area fraction of Bcl-2. Results are introduced as mean ± standard error. The *p* value is cited as letters (different letters mean a significant difference).

**Figure 8:**
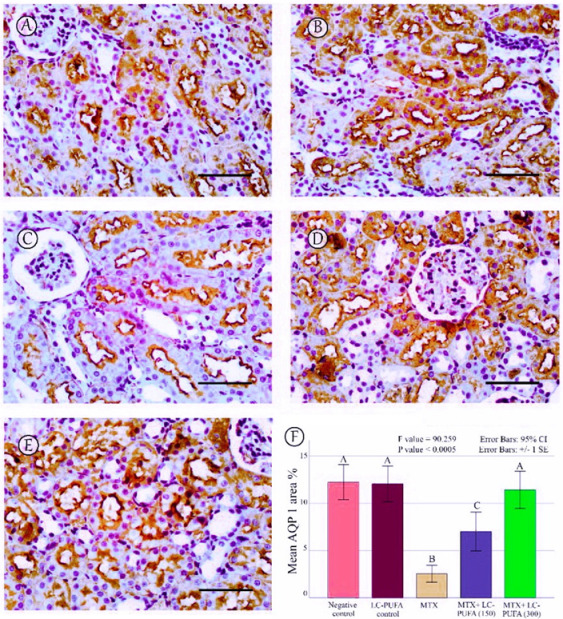
AQP1 immunostaining; (**A**): Saline group; (**B**): LC-PUFA group; (**C**): MTX group; (**D**): MTX + LC-PUFA 150 group; (**E**): MTX + LC-PUFA 300 group. Results revealed a moderate expression in the proximal tubules’ epithelium of the control and LC-PUFA group rats. The expression was reduced in the MTX-treated rats. However, the renal tubular epithelium of MTX + LC-PUFA 150 and MTX + LC-PUFA 300 groups showed increased AQP1 expression. Scale bar = 50 µm. (**F**) area percentage of AQP1. Results are introduced as mean ± standard error. The *p* value is cited as letters (different letters mean a significant difference).

**Figure 9:**
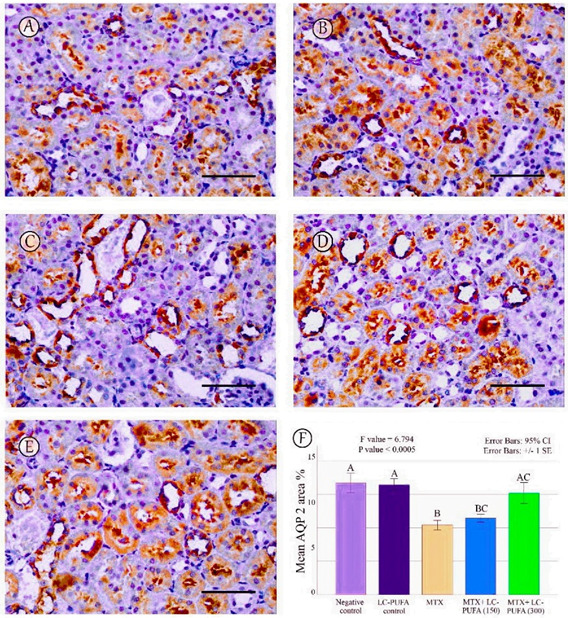
AQP2 immunostaining; (**A**): Saline group; (**B**): LC-PUFA group; (**C**): MTX group; (**D**): MTX + LC-PUFA 150 group; (**E**): MTX + LC-PUFA 300 group. Results revealed a moderate expression in the cytoplasm and apical membranes of the collecting duct epithelium of the saline control, LC-PUFA control, and MTX groups. In the latter, the expression was redistributed mainly to apical membranes. However, the renal tubular epithelium of MTX + LC-PUFA 150 and MTX + LC-PUFA 300 groups showed restoration of the normal distribution of AQP 2 levels. Scale bar = 50 µm. (**F**) area percentage of AQP2. Results are introduced as mean ± standard error. The *p* value is cited as letters (different letters mean a significant difference).

The results of the immunohistochemical analysis of NF-ĸB, BAX, and BCL-2 are demonstrated in Figure 5, Figure 6, Figure 7, Figure 8 and Figure 9. Immunohistochemical staining for NF-ĸB and BAX revealed minimal positive staining in the control and LC-PUFA groups. Moreover, strong reactions for NF-ĸB and BAX were found in the cytoplasm of the epithelium of glomeruli and tubules of MTX-treated rats (Figure 5A–C and Figure 6A–C). On the other hand, there was a weak focal reaction in the tubular epithelium of the MTX + LC-PUFA (150 mg/kg) and (300 mg/kg) groups (Figure 5D,E and Figure 6D,E). Immunohistochemical staining for Bcl-2 of the control and LC-PUFA groups showed moderate expression in the cytoplasm of the renal tubular epithelium (Figure 7A,B), whereas expression was weak in the MTX group (Figure 7C). However, the MTX + LC-PUFA (150 mg/kg) + MTX and (300 mg/kg) groups restored the moderate expression of Bcl2 (Figure 7D,E). Immunohistochemical expression of AQP1 of the control and LC-PUFA groups showed moderate expression in the cytoplasm and membrane of the proximal tubules in the renal tubular epithelium (Figure 8A,B), whereas expression was mild in the MTX group (Figure 8C). However, MTX + LC-PUFA (150 mg/kg) and MTX + LC-PUFA (300 mg/kg) restored the moderate expression of AQP1 (Figure 8D,E). Immunohistochemical expression of AQP2 of the control and LC-PUFA groups showed moderate expression in the cytoplasm and apical membrane of collecting ducts in the epithelium (Figure 9A,B), while MTX showed moderate expression for AQP2 but was redistributed mainly to the apical membranes of the collecting ducts (Figure 9C). However, MTX + LC-PUFA (150 mg/kg) and MTX + LC-PUFA (300 mg/kg) restored the normal distribution of AQP2 (Figure 9D,E). The low power (×100 magnification) for BAX, Bcl-2, AQP1 and AQP2 are demonstrated in Figures S2–S5, respectively.

### 2.5. Effect of LC-PUFA on Morphometric Analysis of Immunohistochemical Findings of NF-ĸB, BAX, Bcl2, AQP 1 and AQP2

The percentage of NF-kB, BAX, Bcl-2, AQP1, and AQP2 immunopositive area and the optical density of AQP1 exhibited a significant difference (*p* < 0.0005) among the study groups; however, the optical density of AQP2 showed an insignificant difference among the studied groups (*p* = 0.303). The Tukey post-hoc tests showed an insignificant difference between the saline control and LC-PUFA groups. It also showed a significant increase in NF-kB, and BAX, with a significant reduction in the expression of the Bcl-2, AQP1, AQP2 area percentage and AQP1 optical density in the MTX group, as compared to the saline and LC-PUFA control groups, with an insignificant difference in AQP2 optical density. There was a significant reduction in NF-kB and BAX, and a significant increase in Bcl-2, AQP1 area percentage, and optical density in the MTX + LC-PUFA 150 and MTX + LC-PUFA 300 groups, when compared with the MTX group, except for AQP2. Moreover, there was a significant difference between the MTX + LC-PUFA300 group and MTX + LC-PUFA 150 group in all the studied parameters except for AQP2. There was an insignificant difference between MTX + LC-PUFA 300 group and the saline and LC-PUFA control groups (Figure 5, Figure 6, Figure 7, Figure 8 and Figure 9).

## 3. Discussion

Methotrexate (MTX) is a potent anticancer and immunomodulatory agent. However, MTX causes toxicity in different organs, and because MTX is mainly excreted by the kidneys, nephrotoxicity is a common adverse effect of MTX [28,29,30]. Excretion of toxins by the kidney is mainly carried out by the proximal convoluted tubules; on the other hand, the proximal tubules are the target for many drugs, such as MTX [31]. This work was designed to find a therapy that protects against MTX-related renal damage. We assumed that an omega-3 long-chain polyunsaturated fatty acid (LC-PUFA) supplement can prevent nephrotoxicity. 

In the present study, MTX-induced nephrotoxicity was associated with disturbed renal function tests, elevated serum creatinine, and urea, and with induction of apoptosis as noticed through upregulation of the apoptosis marker BAX and downregulation of the anti-apoptosis marker BCL-2. We also determined that MTX could induce an inflammatory response, as seen through activation NF-kappaB, similar to the findings of Younis et al. [32] who reported the cytotoxic effect of MTX, causing cell death. In addition, MTX caused renal oxidative stress as marked by increased renal MDA, NO, and decreased renal GSH, consistent with the previous reports [29]. 

In the present study, LC-PUFA administration restored the renal function tests, suppressed the oxidative stress, and attenuated the apoptotic process induced by MTX with a reduction in BAX expression and upregulation in the expression of BCL-2, consistent with the previous findings of Li et al. [26], who concluded that maresin-1, a derivative from docosahexaenoic acid, showed anti-inflammatory, antioxidant, and antiapoptotic effects against lipopolysaccharide-induced acute kidney injury by suppressing NF-kappaB. This is also coincident with the findings of suppression of apoptosis and the renoprotective effect of LC-PUFA after gentamicin intoxication has been proven [33]. Furthermore, the cytoprotective effect of LC-PUFA is consistent with the findings of Elsayed et al. [24], who reported the antiapoptotic effect of LC-PUFA against MTX-induced splenic injury, as noticed by the decreased reaction of caspase-3 and increased reaction of Bcl-2. The findings are similar to the results of Koppelmann et al. [25] who proved the antiapoptotic and anti-inflammatory activities of LC-PUFA against intestinal damage, induced by MTX in a cell line and a rat model, showing reduced injury, enhanced repair, and decreased apoptosis, as indicated by decreased BAX and increased BCL-2 expressions with a decrease in NF-kappaB in the intestinal mucosa. 

Aquaporins (AQPs) are transmembrane water channel proteins. They play vital roles in bacteria, plants, and mammalian organs e.g., the eyes, lungs, brain, as well as in the kidneys. Untill now, 13 types of AQPs have been discovered in mammalian species. Some AQPs show permeability only to water, while others allow glycerol, urea, and other solutes to pass. The kidney has seven types of AQPs present in the different parts of nephrons. In the kidney, AQPs regulate urine concentration by kidney, osmolarity, and volume of fluid [12].

The main site for absorption of the fluid filtered by glomeruli is the proximal convoluted tubules. The apical and basolateral membranes of the epithelial lining of the proximal convoluted tubule, the membranes of the descending limb of Henle, and the endothelial lining of the descending vasa recta of the outer medulla all express AQP1. They are responsible for the countercurrent multiplication system controlling urine concentration [13]. Knocking out of AQP1 did not cause the death of the mice; however, they were more susceptible to intense dehydration. Moreover, they lost the ability to perform appropriate urine concentrations [34]. We found that MTX-induced nephrotoxicity is associated with suppression of the expression of aquaporin 1, similar to the results of Khoshnoud et al. [15] and Türk et al. [17], and this may account for the proximal tubules’ damage, as in the work of Kandemir et al. [31], who reported that downregulation of AQP1 levels was associated with damage of proximal tubules first. Interestingly, LC-PUFA groups showed restoration of AQP1 expressions in a dose-dependent manner.

On the other hand, AQP2, expressed in both the connecting tubule and collecting duct, is responsible for water conservative function under the control of vasopressin. The redistribution of AQP2 towards the apical membranes of principal cells of the collecting ducts regulates water excretion [14]. 

We assessed the expression of AQP2 in the MTX group by immunohistochemistry and detected its redistribution from the cytoplasm of collecting ducts’ principal cells to their apical membranes; however, there was no difference in the optical density of AQP2, as compared to the saline and LC-PUFA control groups, consistent with the results of Severin and Torres [16] who reported similar findings; AQP2 expression in kidney homogenate of MTX group was similar to the control group, with a concentration of AQP2 in apical membranes, suggesting a redistribution of AQP2 which may cause altered water handling. This redistribution occurs in response to vasopressin, as a mechanism regulating water absorption [14]. Interestingly, we report for the first time that LC-PUFA could restore AQP2 expressions in a dose-dependent manner.

## 4. Materials and Methods

### 4.1. Sample Size Estimation

To minimize the use of animals, the sample size was designed by the G*Power program (Version 3.1.9.2, by Franz Faul, Kiel, Germany), as demonstrated by Faul et al. [35]. Based on a previous study by Soliman et al. [36], we proposed that the means for the five groups would be 0.7, 0.9, 0.9, 1.2, and 1.8 for NFKB, 0.8, 0.9, 0.9, 1, and 1.8 for BAX, and 11, 26, 26, 29, and 33 for BCL-2, respectively. Supposing that the standard deviation (SD) within each group is 0.15 for NFKB, 0.3 for BAX, and 0.5 for BCL-2, effect size (f) would be 2.5647 for NFKB, 1.2184 for BAX, and 14.9131 for BCL-2. Based on this proposal, samples sizes of 10, 20, and 10 for NFKB, BAX, and BCL-2, respectively, attain 95% power to notice these effect sizes with an alpha level of 5%, taking into consideration the smallest effect size (1.2184), when using one-way ANOVA test within the five groups. Thus, we estimated the sample sizes as four rats per group. The total sample of 20 attains a power of 95% when utilizing the F-Test, targeting a significance level of 0.050. 

### 4.2. Ethical Statement

The treatment of animals in the experiment followed the standard guidelines for the care and use of laboratory animals and was approved by Mansoura University Animal Care and Use Committee (MU-ACUC), Egypt (MED.R.22.10).

### 4.3. Animals

Twenty adult male Sprague–Dawley rats weighing 200–250 g, were used. The animals were housed in spacious wire mesh cages in a special room with direct daylight and natural ventilation. The rats had free access to standard rat chow and water.

### 4.4. Research Design

The timeline for the study is demonstrated in Figure 1. The animals were divided into five groups (four rats/each), as follows: Group 1 (Saline control) received saline once daily via oral gavage, for five days; Group 2 (LC-PUFA control) was given LC-PUFA (EPA and DHA, Novell Pharma, Egypt), 300 mg/ kg, once daily via oral gavage for five days; Group 3 (MTX) was given saline via oral gavage, once daily for five days and an intraperitoneal injection of MTX 20 mg/kg (Pharmachemie BV, Haarlem, Noord-Holland, The Netherlands) once, at the beginning of the third day of the experiment; Groups 4 and 5 (MTX + LC-PUFA 150 and MTX + LC-PUFA 300, respectively: received LC-PUFA 150 or 300 mg/kg, respectively, and received an intraperitoneal injection of MTX once at the beginning of the third day of the experiment. The rats received LC-PUFA (EPA: DHA); in a ratio of 3:2, which was reported to be most the efficient ratio [37,38,39]. Three days following the injection of MTX, the rats were subjected to anaesthesia by inhalation of ether, and then cervical dislocation was performed for sacrification [40]. Body weights were recorded at the beginning and at the end, and the weight gain was calculated as a percentage of initial weight. Kidneys were excised, washed, and then dried.

### 4.5. Blood Sampling

The rats were subjected to overnight fasting at the end of the experiment. The blood samples were collected from the hearts of rats and put in EDTA-free tubes. The serum was separated and subsequently stored at −20 °C until the time of analysis.

### 4.6. Evaluation of Renal Function Tests

The sera of the rats were examined for the detection of the kidney function tests by colorimetric assay using urea and creatinine detection kits obtained from Bio-Diagnostic Co. (Dokki, Giza, Egypt), and following the manufacturer’s instructions.

### 4.7. Preparation of Tissue Homogenates

Kidney samples were homogenized as described by Elsayed et al. [24], and the supernatant was removed and stored before being used for the detection of oxidative stress markers.

### 4.8. Estimation of Oxidative Stress Markers

Kidney tissue supernatants were used to assess reduced glutathione (GSH), nitric oxide (NO) and malonaldehyde (MDA) levels using detection kits (Bio-Diagnostic company, Egypt), based on previously described methods [41,42,43]. 

### 4.9. Histopathological Examination of the Kidney

Parts of each kidney were excised, washed, dried, and then fixed in formaldehyde (10%) and implanted into paraffin [8], to assess the histopathological changes. Then, 8 μm thick transverse sections were stained using hematoxylin and eosin (H&E). Semiquantitative histopathological scoring for the renal damage was performed. Shrinkage of the glomeruli, dilatation of the Bowman’s capsule, tubular dilatation, tubular cast, inflammatory cell infiltrations, and congestion of renal blood vessels were evaluated and were graded as follows: 0, less than 5%; 1, 5–33%; 2, 34–66%; and 3, over 66%.

### 4.10. Immunohistochemistry 

The immunoperoxidase method was performed on three μm thick renal cortical transverse sections [44,45,46]. In brief, the slides were deparaffinized and endogenous peroxidase was blocked. The kidney sections were supplemented with hydrogen peroxide and 0.3% methanol, for 10 min at room temperature, followed by heating of the sections at 95 °C in 10 mM citrate buffer for 10 min to enhance antigen retrieval. The sections were then left for one hour to cool. The sections were kept with the primary antibodies of NF-kB; a proinflammatory marker, BAX; an apoptosis marker, Bcl-2; an antiapoptotic marker, AQP1 and AQP2, overnight at 4 °C. Table 2 demonstrates the details of the antibodies and their dilutions. The slides were then incubated for 30 min with a mouse–rabbit polydetector (BSB 0268, Bioscience). For the reagent (no primary antibody) control, phosphate-buffered saline (PBS), was added as a substitute for the primary antibody. Finally, washing, dehydration, and examination of the sections was carried out [47]. Dark brown areas, on a blue background, demonstrate positive staining. Antigen localization was mainly cytoplasmic for BAX, Bcl-2, cytoplasmic and nuclear for NF-kB, and cytoplasmic and membranous for AQP1 and AQP2.

### 4.11. Morphometric Analysis

The ImageJ software version 1.52a [48] and Fiji ImageJ software [49] were utilized. We quantified the percentage of NF-ĸB, BAX, and BCL-2, AQP1 and AQP2 immunopositive area, and AQP 1 and 2 optical densities (in ×400). 

### 4.12. Statistical Analysis

The SPSS software was utilized to analyze the results. One-way ANOVA, followed by post-hoc Tukey tests, were utilized to compare the quantitative normally distributed data. The data that are not normally distributed were presented as median and interquartile range) and a Kruskal–Wallis H test was used to compare them. Significant *p* values are considered if ≤0.05.

## 5. Conclusions

We determined that LC-PUFA could restore the renal function tests for creatinine and urea and could normalize the levels of renal oxidative stress markers. Furthermore, LC-PUFA could attenuate the histopathological changes induced by MTX and could decrease BAX, NF-kB, and increase BCL-2 as well as AQP 1 expressions, and could restore the normal distribution of AQP2. Therefore, LC-PUFA can be considered a good adjuvant to MTX to prevent its adverse effects on kidneys through its antioxidant, antiapoptotic effect, and anti-inflammatory effects, and due to its role in the restoration of the expression of AQPs 1 and 2.

## 6. Study Limitations

The animals, species as well as sex differences in the inflammatory response may cause different results. Furthermore, the effect, related to multiple doses and long-term use of MTX and LC-PUFA, may cause differences in results. Thus, we recommend further studies on the different constituents of LC-PUFAs, namely docosahexaenoic acid (DHA), alpha-linoleic acid, and eicosapentaenoic acid (EPA), separately to investigate which one may be more effective. Furthermore, we studied the role of MTX and LC-PUFA on AQPs 1 and 2 only; thus, we recommend studying their effects on other AQPs.

## Figures and Tables

**Table 1:** Effect of LC-PUFA on the histopathological scoring for renal damage.

	Saline Control (n = 4)	LC-PUFA Control (n = 4)	MTX (n = 4)	MTX + LC-PUFA150 (n = 4)	MTX + LC-PUFA300 (n = 4)	H Value	*p* Value
Shrinkage of glomeruli	0(0–0) A	0(0–0) A	2(1–2) B	1(1–1) BC	1(0–1) C	64.778	<0.0005
Dilatation of Bowman’s capsule	0(0–0) A	0(0–0.75) A	2(1.25–2) B	1(1–1) BC	1(0–1) AC	66.029	<0.0005
Tubular dilatation	0(0–1) A	0(0–0) A	2(1.25–2) B	1(1–1) BC	1(0–1) AC	63.082	<0.0005
Tubular cast	0(0–0.75) A	0(0–0) A	2(1.25–2) B	1(1–2) BC	1(0–1) AC	62.767	<0.0005
Inflammatory cell infiltrations	0(0–1) A	0(0–0.75) A	2(1.25–2) B	1(1–1) BC	1(0.25–1) AC	60.135	<0.0005
Congested blood vessels	0(0–0.75) A	0(0–0) A	2(1.25–2) B	1(1–1) BC	1(0–1) AC	64.346	<0.0005
Score	1(1–2) A	1(0–2) A	10(10–11) B	7(7–7.75) BC	4(4–5) C	91.765	<0.0005

Results are tabulated as median and interquartile range. The *p* value was determined by the Kruskal–Wallis H test; different letters = statistically significant difference. Significant *p* values (≤0.05).

**Table 2:** Primary antibodies applied for immunohistochemistry.

Name	Cat. Number	Source and Clonality	Dilution
NF-kB	ABclonal A19653	Rabbit monoclonal	1/100
BAX	ABclonal A12009	Rabbit polyclonal	1/100
Bcl-2	Genemed 60-0005-7	Mouse monoclonal	1/100
AQP1	Servicebio GB11310-1	Rabbit polyclonal	1/500
AQP2	Servicebio GB112259	Rabbit polyclonal	1/400

## Data Availability

All data are presented in the article.

## Supplementary Materials

The following supporting information can be downloaded at: https://www.mdpi.com/article/10.3390/ijms232112794/s1.
