# Supplementary material for: Methotrexate-Induced Alteration of Renal Aquaporins 1 and 2, Oxidative Stress and Tubular Apoptosis Can Be Attenuated by Omega-3 Fatty Acids Supplementation
Source: Int J Mol Sci. 2022 Oct 24;23(21):12794. doi: 10.3390/ijms232112794 (PMC9653681; doi:10.3390/ijms232112794)
Supplement: Supplementary file 1 [file ijms-23-12794-s001.zip › ijms-1936181-supplementary.pdf]

H&E *100

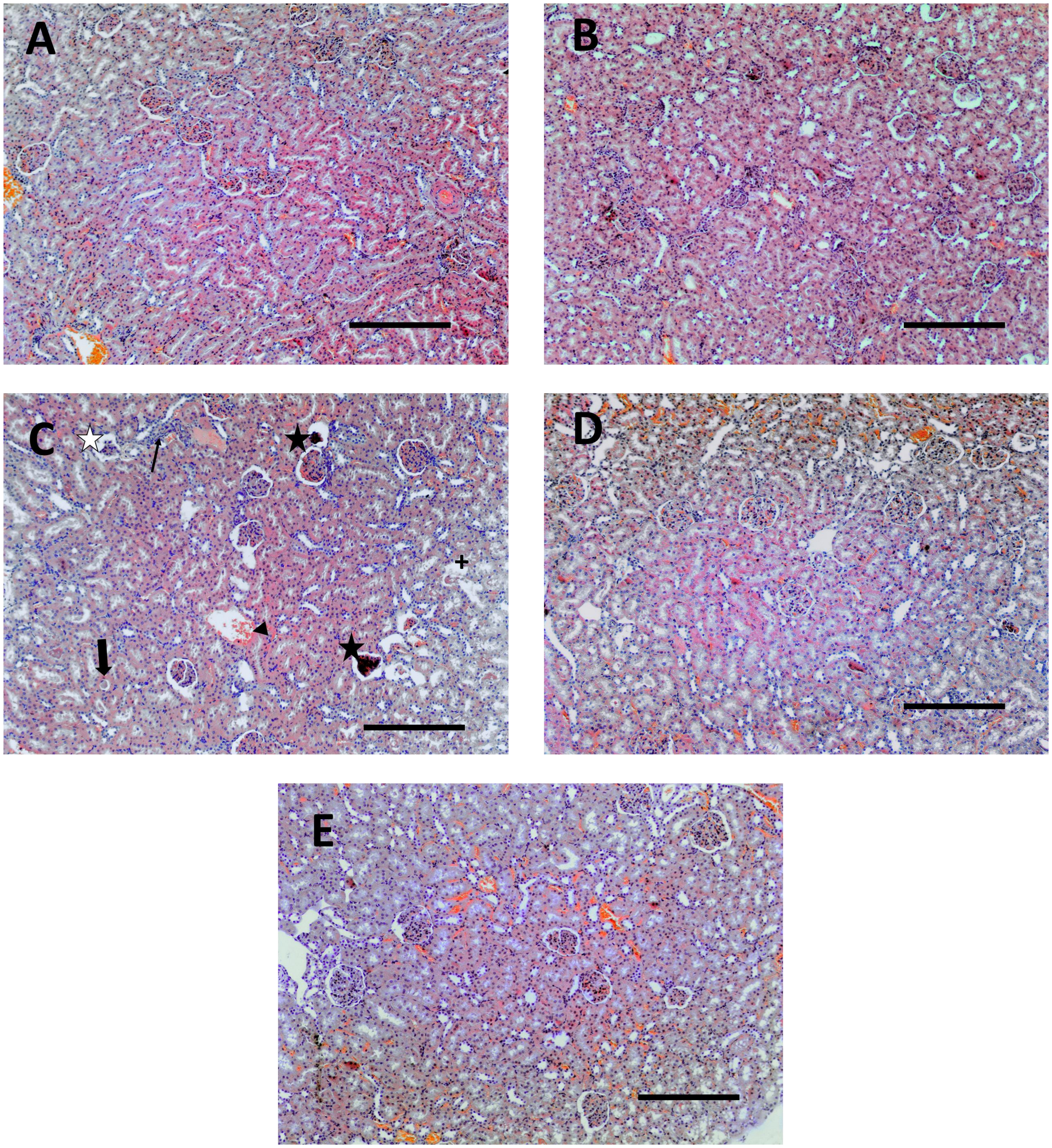

Figure S1. Histopathological changes observed in kidneys. A, B: Sections from Saline and LC-PUFA control groups revealed normal renal glomeruli and tubules. C: MTX group revealed severe damage to the renal tissue, particularly in the renal cortex including shrinkage of glomeruli (star) with dilatation of Bowman's capsule (white star), Tubular dilatation (+) with occasional tubule cast present (thick arrow), inflammatory cell infiltrations (thin arrows), with congestion of renal blood vessels (arrowheads); D: MTX+ LC-PUFA 150 group showed moderate dilatation of tubules with dilatation of Bowman's capsule, inflammatory cell infiltrations with congestion of renal blood vessels; E: MTX+ LC-PUFA 300 group observed maximum improvement in cytoarchitectural changes with no hemorrhage, minimal tubular dilatation, minimal inflammatory cell infiltrations and dilatation of Bowman's capsule (white star). Scale bar = 100 µm.

BAX*100

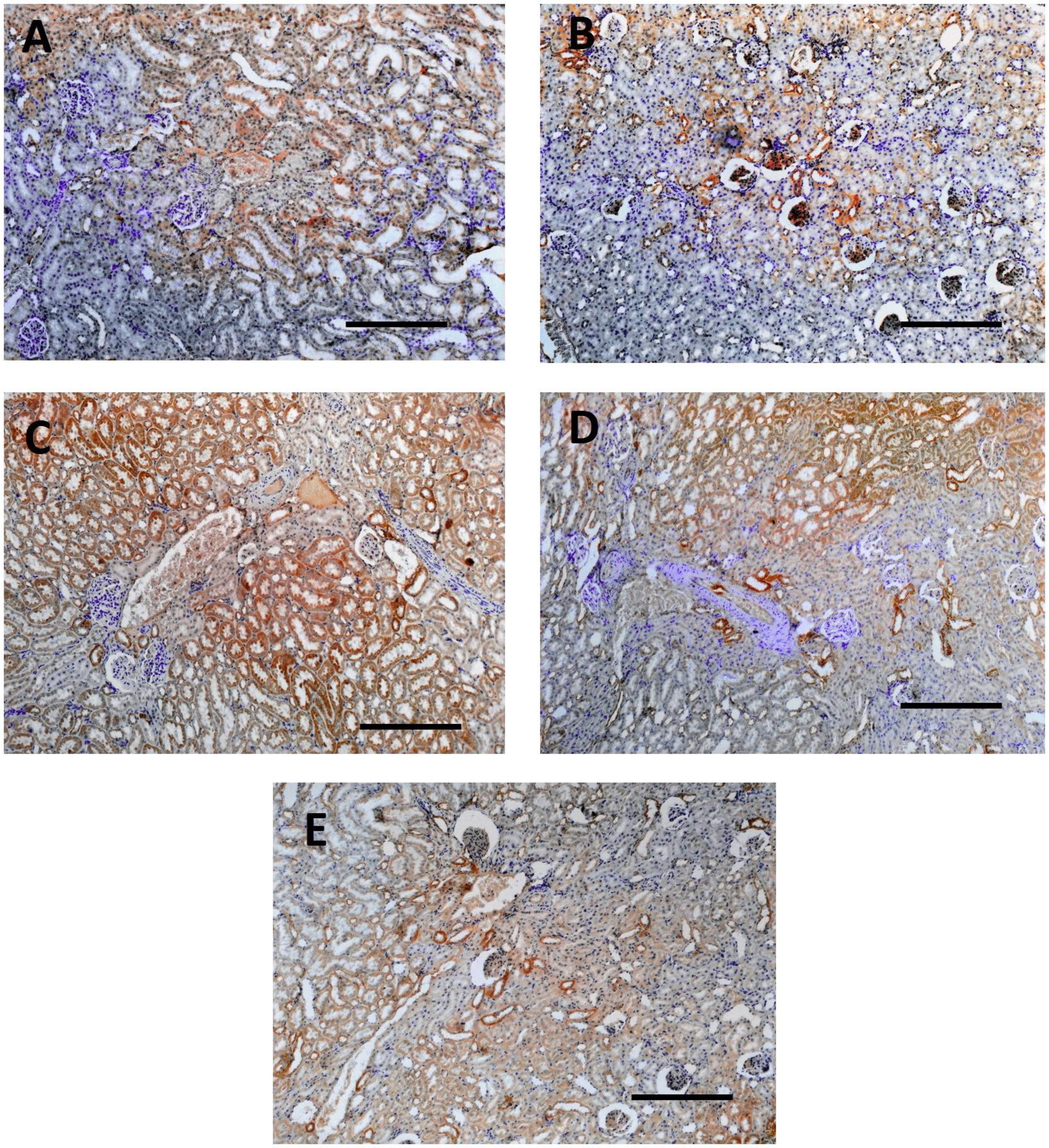

Figure S2: BAX immunostaining. A: Saline group; B: LC-PUFA group; C: MTX group; D: MTX+ LC-PUFA 150 group; E: MTX+ LC-PUFA 300 group. It revealed minimal positive staining in the Saline and LC-PUFA groups. While strong diffuse expression was demonstrated in the MTX-treated rats. Moreover, the epithelium of renal tubules of MTX+ LC-PUFA 150 and MTX+ LC-PUFA 300 groups showed a weak focal reaction. Scale bar = 100 µm.

Bcl-2*100

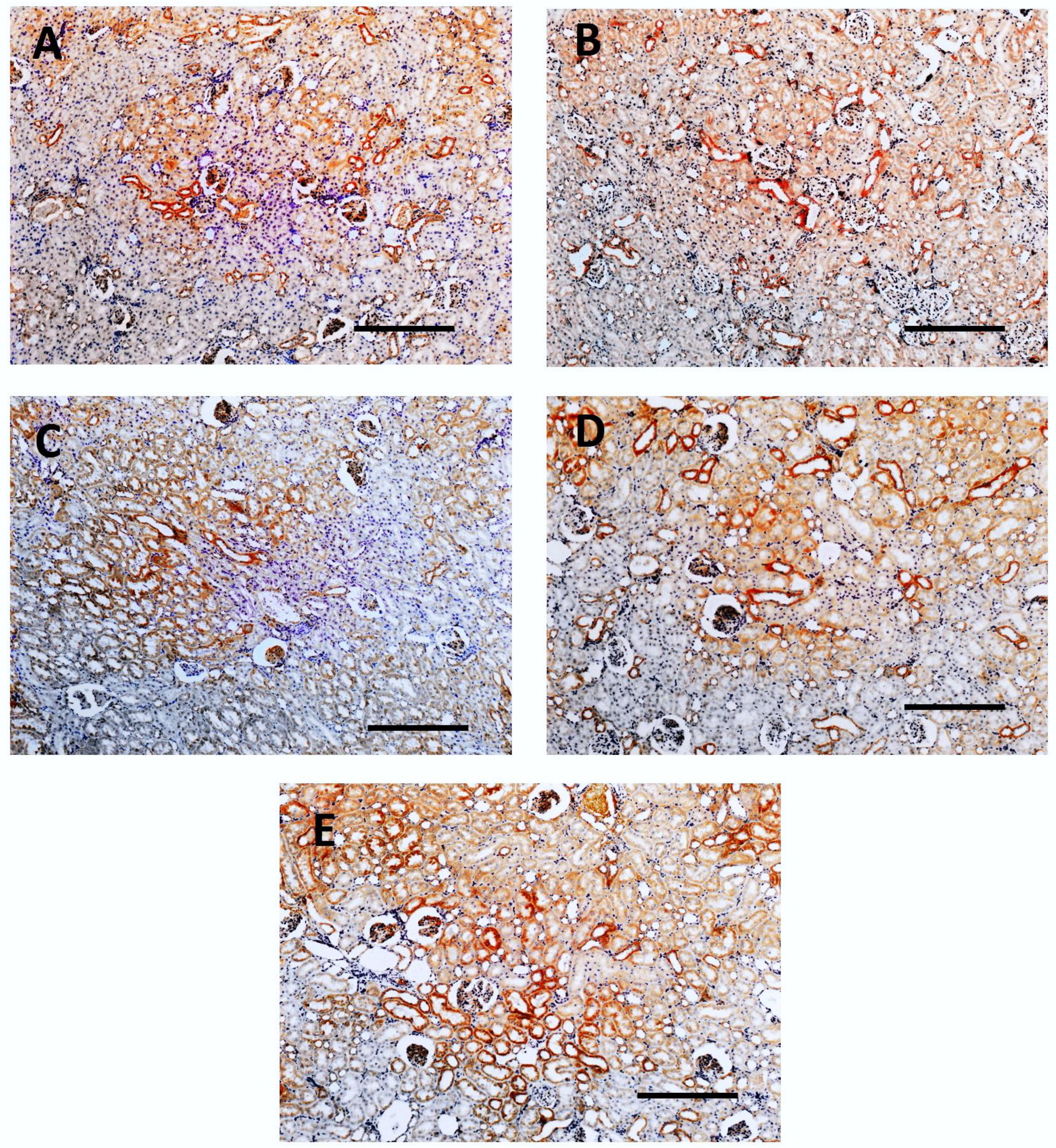

Figure S3: Bcl-2 immunostaining; A: Saline group; B: LC-PUFA group; C: MTX group; D: MTX+ LC-PUFA 150 group; E: MTX+ LC-PUFA 300 group. It revealed a moderate expression in the renal tubular epithelium of the control and LC-PUFA group rats. While the expression was mild in the MTX-treated rats. However, the renal tubular epithelium of MTX+ LC-PUFA 150 and MTX+ LC-PUFA 300 groups showed moderate Bcl2 expression. Scale bar = 100 µm. .

Aquaporin 1 *100

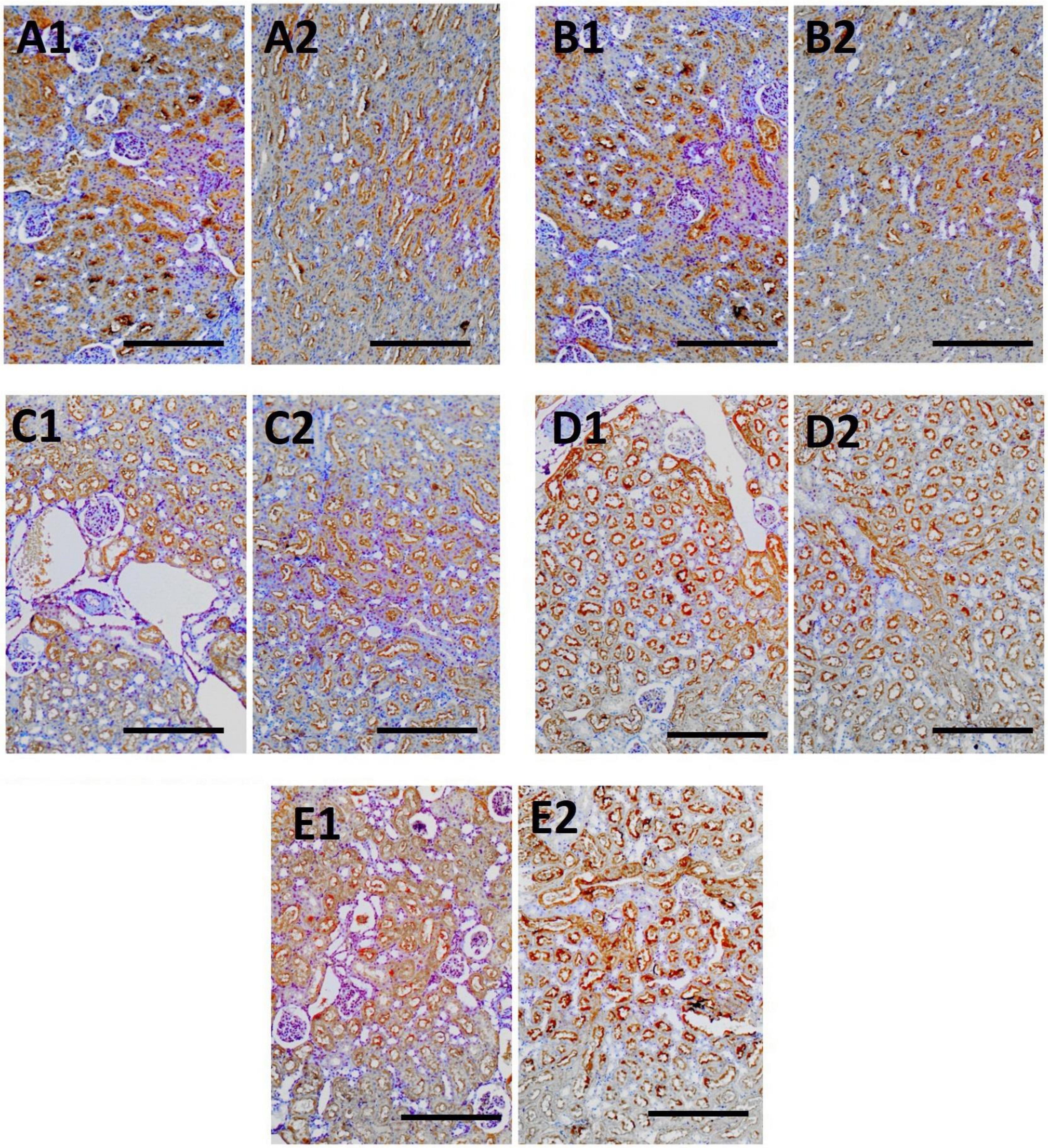

Figure S4: Aquaporin 1 immunostaining; A: Saline group; B: LC-PUFA group; C: MTX group; D: MTX+ LC-PUFA 150 group; E: MTX+ LC-PUFA 300 group. 1: Cortex, 2: Medulla. It revealed a moderate expression in the proximal tubules` epithelium of the control and LC-PUFA group rats. While the expression was reduced in the MTX-treated rats. However, the renal tubular epithelium of MTX+ LC-PUFA 150 and MTX+ LC-PUFA 300 groups showed increased AQP1 expression. Scale bar = 100 μm.

Aquaporin 2 *100

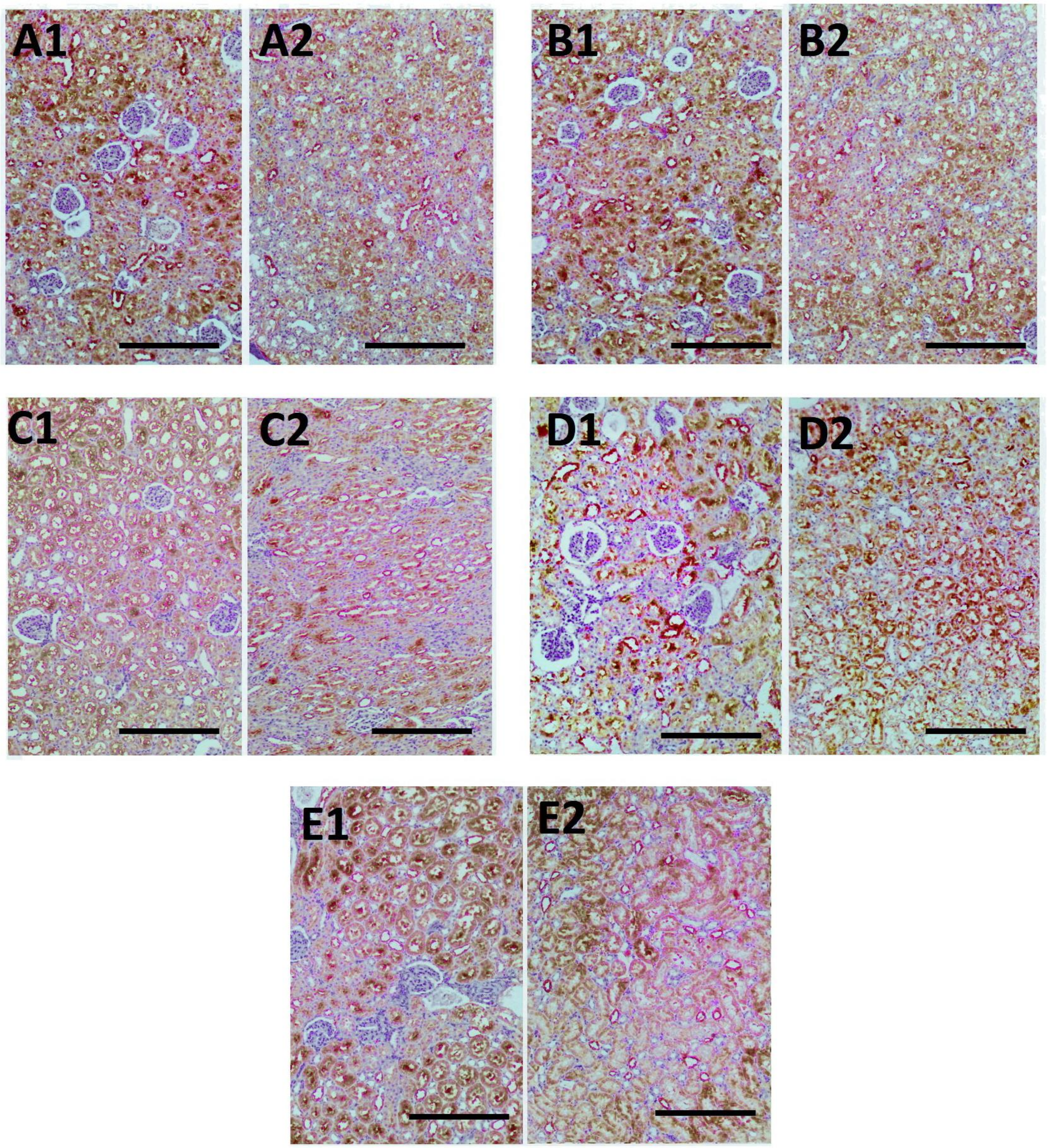

Figure S5: Aquaporin 2 immunostaining; A: Saline group; B: LC-PUFA group; C: MTX group; D: MTX+ LC-PUFA 150 group; E: MTX+ LC-PUFA 300 group. 1: Cortex, 2: Medulla. It revealed a moderate expression in the cytoplasm and apical membranes of collecting duct epithelium of the saline control, LC-PUFA control, and MTX groups. While, in the latter, the expression was redistributed mainly to apical membranes. However, the renal tubular epithelium of MTX+ LC-PUFA 150 and MTX+ LC-PUFA 300 groups showed restoration of the normal distribution of AQP 2 levels. Scale bar = 100 µm.
